# Massage for protrasion of the lumbar intervertebral disci: A systematic review protocol

**DOI:** 10.1097/MD.0000000000020614

**Published:** 2020-07-31

**Authors:** Liu Wu, Bingyan Wan, Mali Xu, Xu Wang, Jin Li, Yang Chen, Wen Gao, Yinhao Feng, Jian Luo

**Affiliations:** a Department of Tuina, Hospital of Chengdu University of Traditional Chinese Medicine, Chengdu; b School of Acupuncture and Tuina, Chengdu University of Traditional Chinese Medicine, Sichuan, China.

**Keywords:** lumbar disc herniation, massage, systematic review

## Abstract

**Background:**

Lumbar disc herniation, which affects the life quality of patients and the social security system adversely, is one of the common spinal diseases. Massage is used as an alternative therapy. Currently, there are no relevant articles for systematic review.

**Methods:**

We will search the randomized controlled trials related to massage therapy lumbar disc herniation to January 2020. The following database is our focus area: the Cochrane Central Register of Controlled Trials (CENTRAL), PubMed, EMBASE, Web of Science, China National Knowledge Infrastructure, Chinese Biomedical Literature Database, and Wan-Fang Database. All published randomized controlled trials in English or Chinese related to massage for lumbar disc herniation will be included.

**Results:**

The results will provide a high-quality synthesis of current evidence for researchers in this subject area.

**Conclusion:**

The conclusion of our study will provide evidence to judge whether massage is an effective intervention in patients the lumbar intervertebral disci.

**PROSPERO registration number:**

CRD42020157303

## 1 Introduction

Lumbar disc herniation (LDH), which affects the life quality of patients and the social security system adversely, is one of the common spinal diseases; its treatment has been paid great attention to in the spine department.^[1]^ There are currently 2 treatments for the disease, one is surgery and the other is conservative treatment. At present, most patients are receiving conservative treatment clinically.^[2–4]^

LDH is also known as a slipped, ruptured, or prolapsed disc. Whatever name is given, it all refers to the medical condition wherein the soft material in the middle of the lumbar disc takes so much pressure that it ruptures. When it occurred, one or more of the nerves in the spine are under pressure. The main symptom of LDH is low back pain and sciatica.^[5]^ In China, about 80% of adults are suffering from low back and leg pain, and 20% of them are diagnosed with LDH.^[6]^ In the United States, there are about 2 million people who are suffering from LDH each year.^[7]^ According to statistics from the World Health Organization, LDH has become one of the most important causes of disability as expressed in disability-adjusted life years both in developed and developing countries. As a result of the significant financial and social burdens associated with LDH, many researches have focused on the identification of effective treatments. Complementary and alternative medicine is widely advocated to face the increasing demand for nonpharmacological approaches.^[8]^ LDH results in dermatomal radicular pain associated with paresthesia and weakness of the lower extremities. The pattern of pain experienced by the patient depends on the level and location of the herniation. Up to 95% of LDHs occur at the L4–L5 and L5–S1 levels.^[9]^

Massage, as any systematic form of touch or manipulation performed on the soft tissues of the body to provide comfort and promote health,^[10–13]^ has become popular in the United States and the rest of the world in recent decades. It has also been recommended by the Chartered Society of Physiotherapy for the management of various pain-related conditions, especially those of musculoskeletal origin,^[14]^ such as neck pain, low back pain, headache, and migraine.^[15–18]^ This is supported by numerous systematic reviews of a large number of randomized controlled trials (RCTs).^[19,20]^ Between 2002 and 2007, the 1-year prevalence of use of massage by the US adult population increased from 5% (10.05 million) to 8.3% (18.07 million), and massage belongs to one of the most popular complementary and alternative medicine (CAM) therapies in the United States.^[20]^ The increased use brings attention to the safety and quality of the modality.

Currently, there is still a lack of evidence-based medical evidence for the treatment of LDH. Therefore, it is necessary to review it and provide evidence for clinicians

## 2 Methods

### 2.1 Study registration

The systematic review protocol has been registered in PROSPERO. The registration number: CRD42020157303, the consent of this protocol report is based on the preferred reporting items for systematic reviews and meta-analyses protocols (PRISMA-P) statement guidelines.^[21]^

### 2.2 Inclusion criteria for study selection

#### 2.2.1 Type of study

We will include articles related to massage therapy of patients with LDH. Due to language restrictions, we will search for articles in English and Chinese in order to get a more objective and true evaluation, all articles included are randomized controlled trial (RCT) type articles.

#### 2.2.2 Type of participant

All patients with LDH will be included regardless of sex, age, race, education, and economic status. Pregnant women, postoperative infections, psychopaths, patients with severe cardiovascular and liver and kidney diseases will not be included.

#### 2.2.3 Type of intervention

Massage therapy including tuina and manipulation while other traditional Chinese therapies such as acupuncture, moxibusition, cupping, and traditional Chinese medicine will be excluded. We will compare the following interventions: treatments other than massage (e.g., usual or standard care, placebo, wait-list controls).

#### 2.2.4 Type of outcome measure

Primary outcomes: The effectiveness and safety of massage patients LDH. Secondary outcomes: Economic index (outcome indicators from the beginning of treatment to the end of treatment).

### 2.3 Data sources

We will perform a systematic electronic search of PubMed, Embase, the Cochrane Library, Chinese Biomedical Literature Database (CBM), Chinese National Knowledge Infrastructure (CNKI), and Wan-fang Data Database from the inception to November 2019. About other sources, we also plan to manually search for the unpublished conference articles and the bibliography of established publications.

### 2.4 Search strategy

The search terms on PubMed are as follows: massage (e.g., “ acupoints” or “tuina” or “manipulation”); LDH (e.g., “Herniation of the Lumbar Disks” or “Lumbar Intervertebral Disk Hernia” or “Lumbar Disc Herniation”); randomized controlled trial (e.g., “randomized” or “randomly” or “clinical trial”). Combinations of Medical Subject Headings (MeSH) and text words will be used. The same search term is used in electronic databases in China. These search terms are shown in Table 1.

**Table 1:**
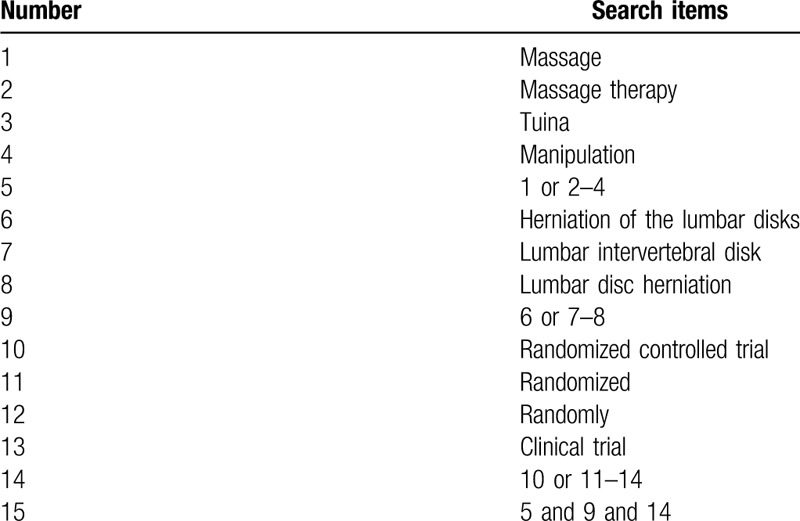
Search strategy for the PubMed database.

### 2.5 Data collection and analysis

#### 2.5.1 Selection of studies

**Figure 1:**
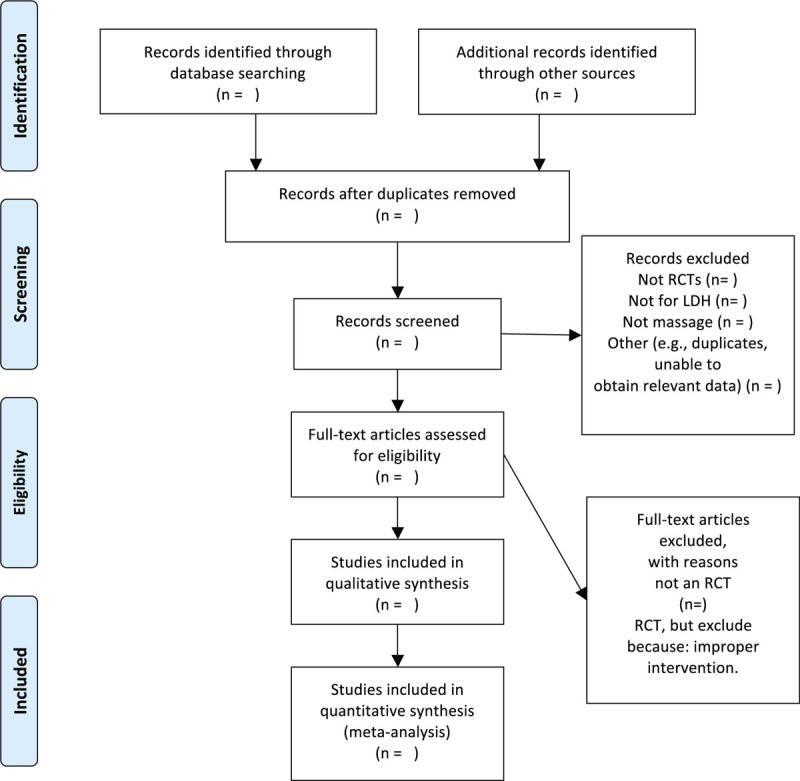
Flow chart of the study.

We chose the PRISMA flow chart to show the process of selecting literature for the entire study (Fig. 1). Before searching the literature, all reviewers will discuss and determine the screening criteria. After the screening requirements are clearly defined, the 2 reviewers (LW and BYW) will independently review and screen the literature. They screened the titles and abstracts of the literature, in order to get qualified studies, and then excluded some duplicate studies or studies with incomplete information. We will also try to obtain the full text, and the obtained literature will be managed by using EndNote software V.X8 (United States). In case of disagreement between the 2 reviewers, discussions will be held with the third author (MLX) for arbitration.

#### 2.5.2 Data extraction and management

The authors will strictly follow the inclusion criteria and select RCT articles related to the topic. Through the analysis of the article, we know participants’ characteristics (height, weight, sex), interventions, outcomes, the study characteristics (press, nationality, journals, research design), adverse reactions, etc. If there is any disagreement between the 2 authors in the literature data extraction, a third article participant (LW) will discuss the decision together. If there is a lack of data in the literature, we will contact the author or publisher as much as possible.

#### 2.5.3 Assessment of risk of bias in included studies

We will use the Cochrane collaborative tool to independently assess the risk of bias in the included studies. We will evaluate the following aspects of the article: sequence generation, assignment sequence hiding, blindness of participants and staff, outcome evaluators, incomplete result data, selective result reporting, and other sources of bias. The risk of bias is evaluated at 3 levels, namely, low risk, high risk, and ambiguity. If the information is vague, we will try to contact the author of the article.

#### 2.5.4 Measures of treatment effect

In this protocol, we will use 95% confidence interval (CI) risk ratio (RR) to rigorously analyze the dichotomous data. And for the continuous data, mean difference (MD) or standard MD (SMD) is used to measure the efficacy of 95% CI.

#### 2.5.5 Unit of analysis issues

We will include data from parallel group design studies for meta-analysis. In these trials, we will collect and analyze individual measurements of each outcome for each participant.

#### 2.5.6 Management of missing data

We will try our best to ensure the integrity of the data. If the included RCT data is not complete, we will try every means to contact the corresponding author of the article, including sending emails or making a phone call. If the corresponding author cannot be contacted, we will remove the experiment with incomplete data. After data integrity is assured, intention analysis therapy and sensitivity analysis will be performed.

#### 2.5.7 Assessment of heterogeneity

For the detection of heterogeneity, the *I*^2^ test will be used to detect the heterogeneity among trials. When the *I*^2^ test value is <50% and *P* value >1, we think there is no heterogeneity between these trials, and when the *I*^2^ test value is >50% and the *P* value is <1, there is significant heterogeneity between these included trials. If significant differences are detected, we will analyze the possible causes of heterogeneity, and then we can use the random effects model.

#### 2.5.8 Assessment of reporting biases

In this analysis, once >10 trials are included, funnel plots could be used to test for reporting bias.

#### 2.5.9 Data synthesis

We will use Review Manager Software (RevMan) V.5.3 (Copenhagen, Denmark) for data analysis and quantitative data synthesis. If there is no finding of statistical heterogeneity, the fixed-effect model is used for data synthesis. If there is significant statistical heterogeneity, we will use the random effect model, and all participants will explore the possible causes from a clinical and methodological perspective and provide a descriptive or subgroup analysis.

#### 2.5.10 Subgroup analysis

Subgroup analysis will be performed to explain heterogeneity if possible. Factors such as different types of control interventions and different outcomes will be considered.

#### 2.5.11 Sensitivity analysis

Based on sample size, study design, heterogeneous quality, methodological quality, and statistical model, sensitivity analysis will be performed to exclude trials with quality defects and ensure the stability of the analysis results.

#### 2.5.12 Grading the quality of evidence

This paper will use the evidence quality rating method to evaluate the results obtained from this analysis. GRADE is generally applied to a large amount of evidence. It has 4 evaluation levels, namely, high, medium, low, and very low. GRADE was used to evaluate the bias, inconsistencies, discontinuities, and inaccuracies of test results. In the context of the system review, quality reflects our confidence in the effectiveness of assessment.^[22]^

#### 2.5.13 Ethical review and informed consent of patients

Ethics and dissemination: The content of this article does not involve moral approval or ethical review and will be presented in print or at relevant conferences.

## 3 Discussion

This systematic review will assess the effectiveness of massage for LDH. There are 4 sections in the review: identification, study inclusion, data extraction, and data synthesis. This review will help the doctors to choose massage as an alternative treatment for LDH patients, and offer the patients more options to relieve their symptoms.

## Author contributions

**Conceptualization:** Liu Wu.

**Data curation:** Jin Li, Bingyan Wan, Wen Gao, Yang Chen.

**Formal analysis:** Bingyan Wan, Yinhao Feng, Mali Xu.

**Resources:** Jian Luo.

**Software:** Liu Wu.

**Writing – original draft:** Liu Wu.

**Writing – review & editing:** Yinhao Feng, Xu Wang, Jian Luo.
